# Multi-parameter MRI based radiomics nomogram for predicting telomerase reverse transcriptase promoter mutation and prognosis in glioblastoma

**DOI:** 10.3389/fneur.2023.1266658

**Published:** 2023-09-26

**Authors:** Ling Chen, Runrong Chen, Tao Li, Chuyun Tang, Yao Li, Zisan Zeng

**Affiliations:** ^1^Department of Radiology, The First Affiliated Hospital of Guangxi Medical University, Nanning, Guangxi, China; ^2^Department of Radiology, Liuzhou Workers Hospital, The Fourth Affiliated Hospital of Guangxi Medical University, Liuzhou, Guangxi, China; ^3^Department of Neurosurgery, Liuzhou Workers Hospital, The Fourth Affiliated Hospital of Guangxi Medical University, Liuzhou, Guangxi, China

**Keywords:** radiomics, nomogram, glioblastoma, telomerase reverse transcriptase, magnetic resonance imaging

## Abstract

**Objective:**

To investigate the clinical utility of multi-parameter MRI-based radiomics nomogram for predicting telomerase reverse transcriptase (TERT) promoter mutation status and prognosis in adult glioblastoma (GBM).

**Methods:**

We retrospectively analyzed MRI and pathological data of 152 GBM patients. A total of 2,832 radiomics features were extracted and filtered from preoperative MRI images. A radiomics nomogram was created on the basis of radiomics signature (rad-score) and clinical traits. The performance of the nomogram in TERT mutation identification was assessed using receiver operating characteristic (ROC) curve, calibration curves, and clinical decision curves. Pathologically confirmed TERT mutations and risk score-based TERT mutations were employed to assess patient prognosis, respectively.

**Results:**

The random forest (RF) algorithm outperformed the other two algorithms, yielding the best diagnostic efficacy in differentiating TERT mutations, with area under the curve (AUC) values of 0.892 (95% CI: 0.828–0.956) and 0.824 (95% CI: 0.677–0.971) in the training set and validation sets, respectively. Furthermore, the predictive power of the radiomics nomogram constructed with the rad-score and clinical variables reached 0.916 (95%CI: 0.864, 0.968) in the training set and 0.880 (95%CI: 0.743, 1) in the validation set. Calibration curve and decision curve analysis findings further uphold the clinical application value of the radiomics nomogram. The overall survival of the high-risk subgroup was significantly shorter than that of the low-risk subgroup, which was consistent with the results of the pathologically confirmed TERT mutation group.

**Conclusion:**

The radiomics nomogram could non-invasively provide promising insights for predicting TERT mutations and prognosis in GBM patients with excellent identification and calibration abilities.

## Introduction

Despite standard treatment, glioblastoma (GBM) is the most common and aggressive primary brain tumor in adults and has high recurrence and mortality rates (1, 2). The 2021 WHO classification system has redefined this entity and separated it into two distinct categories on the basis of its isocitrate dehydrogenase (IDH) genetic makeup (3, 4). The recommendation contributes to the accurate classification and characterization of IDH wild-type GBM (5). As a newly added molecular marker in the classification, telomerase reverse transcriptase (TERT) is of great significance for the diagnosis of GBM. Furthermore, and the presence of TERT promoter mutations has been identified as an important prognostic marker. TERT promoter mutations are reportedly associated with a poorer prognosis (6, 7). GBM with TERT gene mutations typically exhibit more invasive and malignant biological behavior, higher tumor recurrence rate, and poorer survival rate. However, GBM without TERT gene mutations generally have a better prognosis and higher survival rate. Furthermore, TERT gene subtyping can also guide the selection of treatment strategies for GBM (8). GBM with TERT gene mutations may be insensitive to radiation therapy and chemotherapy, requiring more aggressive treatment approaches such as surgical resection and targeted therapy. However, GBM without TERT gene mutations may respond better to conventional treatment protocols, leading to improved treatment outcomes.

Telomeres, which govern the restricted division of normal cells, are reduced with each division of normal cells; however, telomerase in cancer cells may constantly prolong cell division (9). Telomerase comprises an RNA component and reverse transcriptase, which maintain telomere length by adding DNA sequence repeats to chromosome ends to avoid chromosomal shortening during DNA replication (10). Mutations in the TERT promoter region increase gene expression and activate telomerase activity, thus giving tumor cells an infinite potential to proliferate and encouraging tumor development and spread (11). Notably, TERT gene mutations have been considered among the most common genetic alterations. In GBM, the subtypes of the TERT gene are mainly based on genetic variations that regulate its expression levels. Studies have shown that different subtypes of GBM with TERT promoter mutations exhibit distinct molecular characteristics and clinical behaviors (12). The two major subtypes are referred to as TERT-WT (wild-type) and TERT-Mut (mutant). Up to 80% of mutations were in the two hotspots C228T and C250T (13). These mutations lead to excessive expression of the TERT gene, enhancing the unrestricted proliferation and growth capacity of tumor cells. Therefore, TERT promoter mutations can serve as an important biomarker for GBM.

Gene sequencing plays a crucial role in the diagnosis and classification of TERT subtype in GBM patients. However, there are certain limitations and aspects where gene sequencing may not be sufficient. Firstly, GBMs are known for their intratumoral heterogeneity, with different regions of the tumor exhibiting distinct molecular profiles. Gene sequencing performed on a single biopsy sample may not capture the full spectrum of genetic alterations present within the tumor, leading to an incomplete understanding of its molecular characteristics. Secondly, although gene sequencing can identify various genetic alterations, distinguishing between driver mutations (those that contribute to tumor development) and passenger mutations (random genetic changes) can be challenging. Understanding the functional implications of these alterations, such as their impact on cellular pathways or response to targeted therapies, requires additional experimental validation or integration with other imaging data. Furthermore, performing comprehensive gene sequencing can be time-consuming and costly, especially when analyzing large numbers of genes or whole-genome sequencing. These factors may limit the widespread application of gene sequencing in routine clinical practice.

Therefore, for the stratification and characterization of these tumors, it is crucial to rely on non-invasive alternative methods like magnetic resonance imaging (MRI). Recent studies have shown that MRI-based radiomics can provide valuable insights into the biological characteristics and treatment response of gliomas (14–16). By analyzing the texture, shape, and intensity of gliomas in MRI images, researchers can also identify imaging features that are associated with specific genetic mutations. Some studies applied radiomics and identified the TERT promotor genotype in gliomas with an accuracy of over 60%. Nevertheless, most of the previous studies have mainly focused on grades 2–4 gliomas (17–19). Additionally, multi-parameter MRI-based radiomics nomogram in GBM has not been well reported yet. Based on the abovementioned reasons in the present study, we aimed to construct a stable and reliable radiomics model for predicting TERT promoter mutations and prognosis in patients with GBM. To the best of our knowledge, there is a paucity of research on TERT in GBM, and our study also aimed to add a body of knowledge in this area. This approach may enable the development of better management strategies for this devastating disease.

## Materials and methods

### Patients

**Figure 1:**
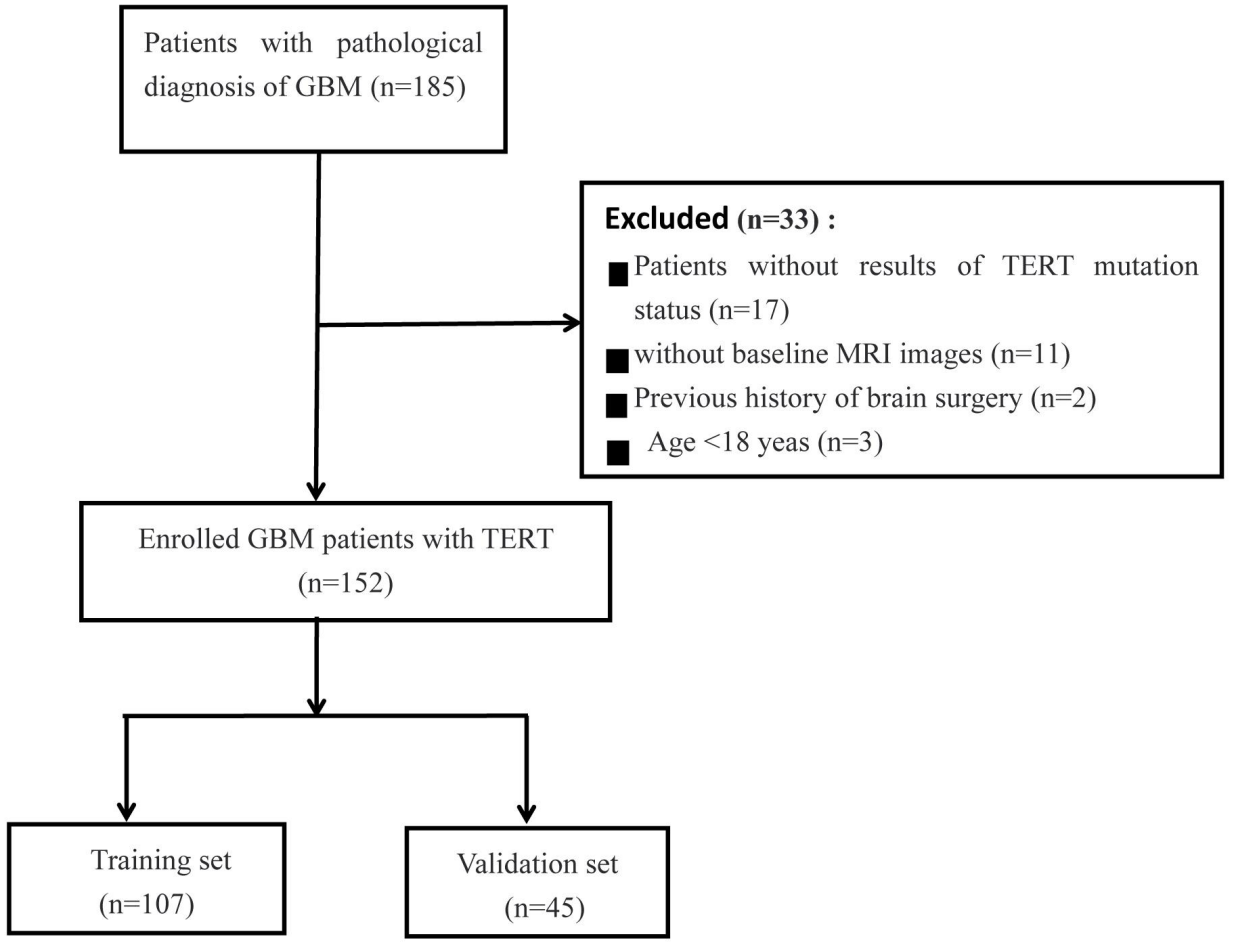
The patient selection flow chart.

This retrospective study was approved by the institutional research ethics review board, and the requirement for obtaining patient consent was waived. In our cohort, 185 patients with GBM patients were included from two centers (Institution I, *n* = 158; Institution II, *n* = 27) between January 2019 and February 2023. All patients underwent resection and genetic testing, and clinical information on various aspects, such as age, sex, overall survival (OS) in months, preoperative Karnofsky Performance Status (KPS) score, and pathological data, was collected from the hospital information system. The MRI images were obtained from the Picture Archiving and Communication Systems (PACS) of the institutes. The inclusion criteria were as follows: (1) Pathological diagnosis consistent with the study; (2) Time elapsed between MRI examination and surgery not exceeding 1 week; (3) No history of surgery or chemoradiotherapy; (4) Available preoperative MRI imaging data; (5) Age > 18 years. The patient selection flowchart is shown in Figure 1.

### MRI protocol

MRI imaging data included axial T2WI, DWI, and ADC sequences obtained on two 1.5 MRI system (GE, Octane, United States; Siemens, Altea, Germany) and two 3.0 T MRI system (Philips, Achieva, Netherlands; GE, Premier, United States). The MRI parameters used are provided in Supplementary material 1.

### Pathological assessment

To determine the status of the TERT promoter mutation, histological analysis was performed for all GBM tissues obtained by surgical resection. Sanger sequencing was used to identify TERT promoter mutations, as shown in this report (20, 21).

### Radiomics process

#### Images preprocessing and segmentation

Herein, T2WI, ADC and DWI DICOM images were imported into the 3D Slicer software (version 5.3.0).^1^ The images were resampled to voxel size of 1 mm × 1 mm × 1 mm, and the gray level was discretized with a bin width of 25. These steps helped reduce the variability caused by differences in scanning parameters and equipment. The volume of interest (VOI) was semi-automatically plotted on T2WI along the tumor margin slice by slice and automatically registered to DWI and ADC images. Tumor segmentation was performed by two neuroradiologists with 10 years of experience in neuroradiology. An interclass correlation coefficient value between 0.75 and 1 indicated good agreement. Any disagreement between the two neuroradiologists was resolved by consensus. The radiomics process is shown in Supplementary material 2.

#### Feature extraction

Radiomics feature extraction was performed by using FeAture Explorer (version 0.3.7)^2^ on Python (3.7.6). We extracted a total of 2,553 (934 × 3) features for each patient. These features could be categorized into five groups, namely shape features (*n* = 14); first-order features (*n* = 18); texture features [gray level co-occurrence matrix (GLCM, *n* = 24), gray level dependence matrix (GLDM, *n* = 14), gray level run length matrix (GLRLM, *n* = 16), gray level size zone matrix (GLSZM, *n* = 16), and neighborhood gray-tone difference matrix (NGTDM, *n* = 5)], wavelet transform (*n* = 744), and Laplacian of Gaussian filter (*n* = 93). A total of 2,553 (934 × 3) features were extracted for each patient. Feature classification and radiomics parameters are shown in Supplementary material 3.

#### Feature selection and signature development

The datasets were randomly divided into two groups (the training set and the validation set) in a 7:3 ratio. Before feature reduction and selection in the training cohort, all extracted features are normalized using Z-score normalization. The Mann–Whitney *U*-test or independent *t-*test was used to assess the relevant features for differentiating TERT mutation status at baseline. Next, three commonly used machine learning algorithms, namely the support vector machine (SVM), random forest (RF), and the least absolute shrinkage and selection operator (LASSO), were compared and the optimal algorithm was chosen for signature construction. A rad-score based on the best classification algorithm was assigned using the coefficient and preserved radiomics features.

A radiomics nomogram combining the rad-score and clinical variables was constructed using multivariate logistic regression analysis. The radiomics nomogram was verified on the validation cohort. The area under the curve (AUC), accuracy, sensitivity, specificity, positive predictive value (PPV), and negative predictive value (NPV) were applied to access the performance of the predictive model. The DeLong test was used to compare the performance of the ROC curves. The calibration curve was used to evaluate the degree of agreement between the predicted probability and observed outcomes across different risk levels. The Hosmer–Lemeshow test was used to evaluate the fit of all models. Decision curve analysis (DCA) was performed to quantify the net benefits under different threshold probabilities in the validation set.

#### Prognosis analysis

All patients were divided into high-risk (predicted TERT mutation-positive) and low- risk (predicted TERT mutation-negative) groups according to their radiomics nomogram risk score. The Kaplan–Meier curve was used to compare the survival analysis results of patients with GBM between pathological diagnosis TERT mutant groups and risk stratification groups using the risk score. The log-rank test was performed to determine differences in survival between these two groups.

### Statistical analysis

SPSS (version 27.0; IBM) and R statistical software (version 4.0.2) were used for statistical analyses. The independent-samples *t*-test or Mann–Whitney U-test was used for continuous variables. The chi-square test or Fisher’s exact test was used for categorical variables. The Kaplan–Meier method was used to assess OS between high-risk and low-risk subgroups, TERT mutant and wild-type subgroups. The survival curves were compared using the log-rank test. A *p-*value of <0.05 was considered indicative of statistical significance.

## Results

### Patient characteristics

The basic clinical characteristics of the GBM patients are showed in Table 1. One hundred and fifty-two patients [67 women, 85 men; mean age, 52.82 ± 11.09 years (range, 27–75 years)] were enrolled in this study. Among these, 72 patients (47.37%) were diagnosed as TERT mutation-positive and 80 patients (52.63%) as TERT mutation-negative. There was no significant difference in sex distribution between the TERT subgroups. However, the age and KPS score were significantly different between the TERT mutation subgroups (*p* < 0.05 for both).

**Table 1:** The basic clinical characteristics of the GBM patients.

Characteristics	Training set (*n* = 104)			Validation set (*n* = 43)		*p*	
	TERT-mt	TERT-wt	*p*	TERT-mt	TERT-wt		*p*
Age (mean ± SD)	57.20 ± 10.00	53.46 ± 9.41	0.035	56.44 ± 10.38	53.08 ± 9.34	0.028	0.569
Male	25	32		13	17		
Sex		0.131				0.011	0.954
Female	23	24		7	6		
KPS (mean ± SD)	3.40 ± 12.72	80.93 ± 12.33	0.026	76.11 ± 10.23	80.80 ± 9.54	0.034	0.073
OS (median [IQR])	16.8 [11.4, 27.1]	28 [19, 34]	0.042	17.2 [12.5, 24]	26 [15, 36.7]	0.045	0.056
TERT mutant		47 (65.28%)		25 (34.72%)			0.042

TERT-mt: Telomerase reverse transcriptase mutant-type; TERT-mt: Telomerase reverse transcriptase wilde-type; KPS: Karnofsky performance status; OS: overall survival; IQR: interquartile range.

### Radiomics feature selection and model construction

**Figure 2:**
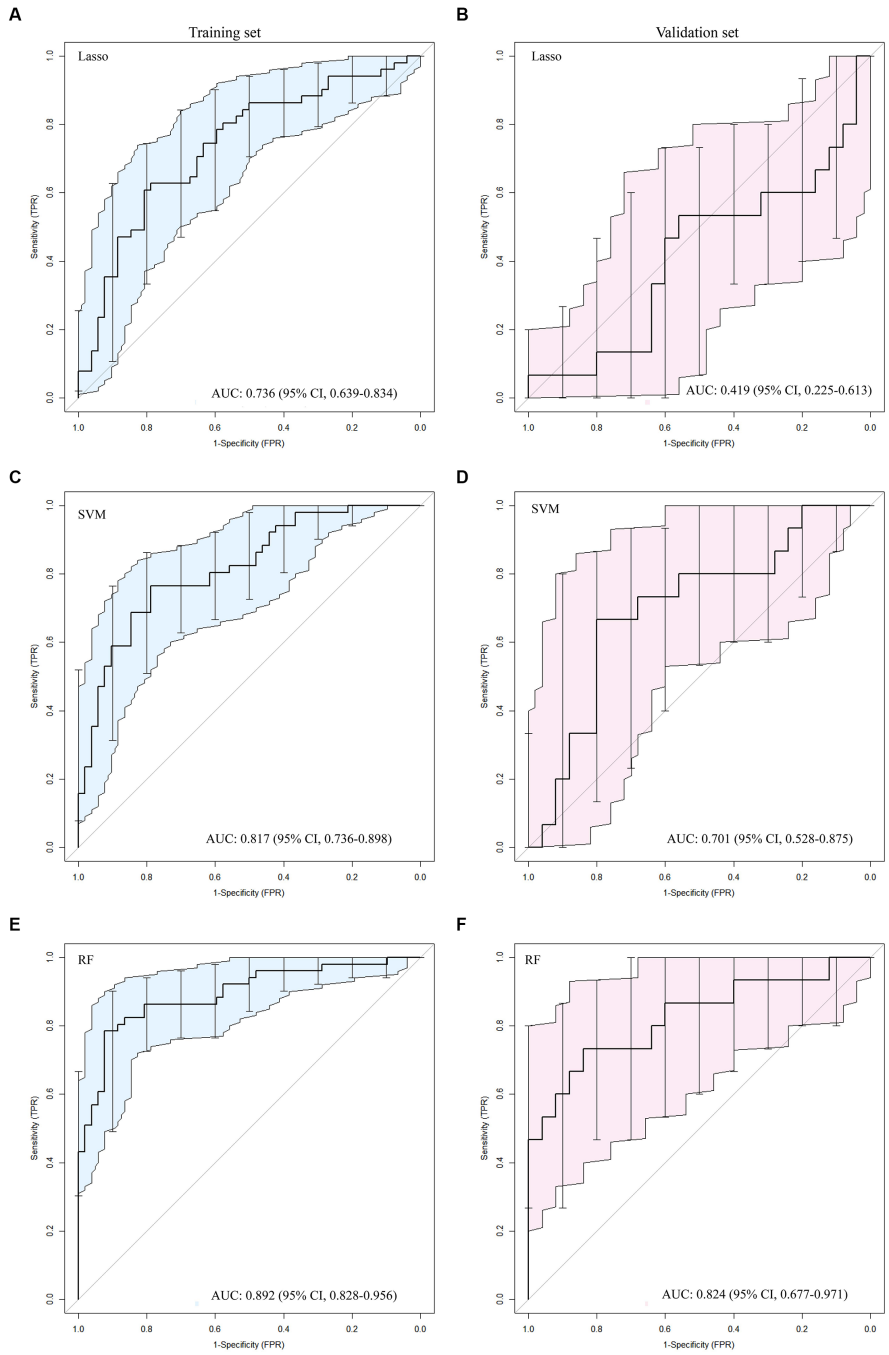
The ROC curves for the Lasso **(A, B)**, SVM **(C, D)** and RF **(E, F)** algorithms were plotted separately for the training and validation sets, respectively.

The ROC curve results for SVM, RF, and LASSO algorithms in the training and validation sets are shown in Table 2 and Figure 2. The result revealed that with the highest diagnostic efficiency for 20 features, the RF algorithm performed the best among the three algorithms, followed by SVM and LASSO algorithms. The AUC values, accuracy, sensitivity, specificity, PPV, and NPV for RF in the training set were 0.892, 0.854, 0.784, 0.923, 0.909, and 0.814 respectively; while in the validation set, they were 0.824, 0.775, 0.733, 0 0.800, 0.688, and 0.833, respectively. In addition, among the 2,832 extracted features, 2,100 features were excluded by baseline analysis, and then, 20 features from among the remaining ones were selected using RF analysis. Finally, the pipeline produced a simpler model of seven features using the “one standard error” rule (22). The features reserved from SVM, LASSO, and RF pipelines are listed in Supplementary material 4. The findings revealed that among the seven most influential features identified by the RF model, four were derived from ADC maps, two from DWI images, and one from T2WI images.

**Table 2:** The AUC, 95% CI, cut-off, accuracy, sensitivity, specificity, PPV, and NPV for LASSO, SVM, and RF model in training and validation sets.

Feature set	AUC	95% CI	Cutoff	Acc	Sen	Spe	PPV	NPV	Tasks
Lasso	0.736	[0.639–0.834]	0.475	0.709	0.627	0.788	0.744	0.683	Training
Lasso	0.419	[0.225–0.613]	0.475	0.475	0.533	0.44	0.364	0.611	Validation
SVM	0.817	[0.528–0.875]	0.468	0.777	0.765	0.788	0.780	0.774	Training
SVM	0.701	[0.816–1.000]	0.468	0.625	0.733	0.56	0.5	0.788	Validation
RF	0.892	[0.828–0.956]	0.560	0.854	0.784	0.923	0.909	0.814	Training
RF	0.824	[0.677-0.971]	0.560	0.775	0.733	0.800	0.688	0.833	Validation

Lasso, The least absolute shrinkage and selection operator; SVM, Support Vector Machine; RF, random forest; AUC, the area under the receiver operator characteristics curve, CI, confidence interval, PPV, positive predictive value, NPV, negative predictive value.

**Figure 3:**
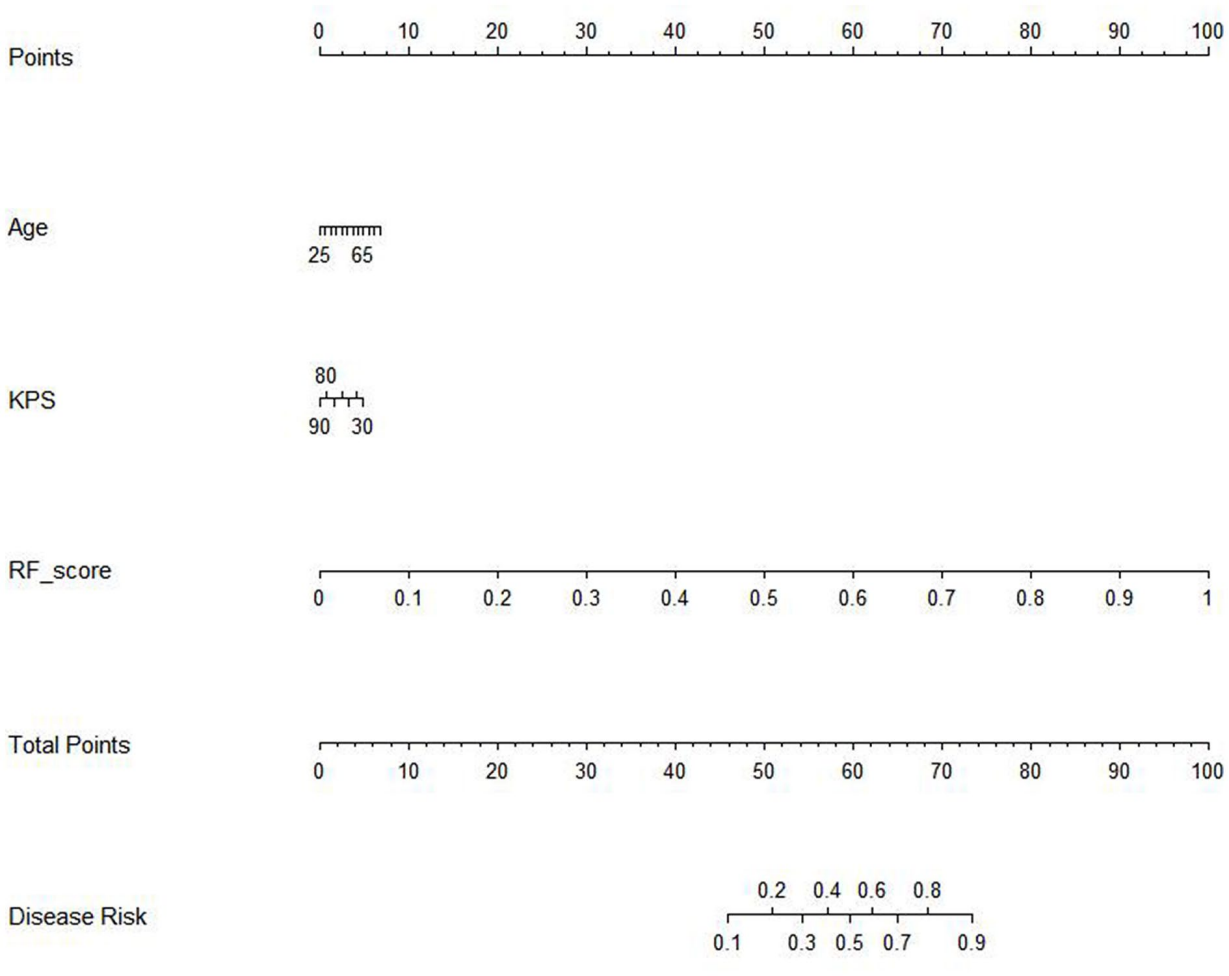
The radiomics nomogram comprised rad-score based on RF and clinical features.

**Figure 4:**
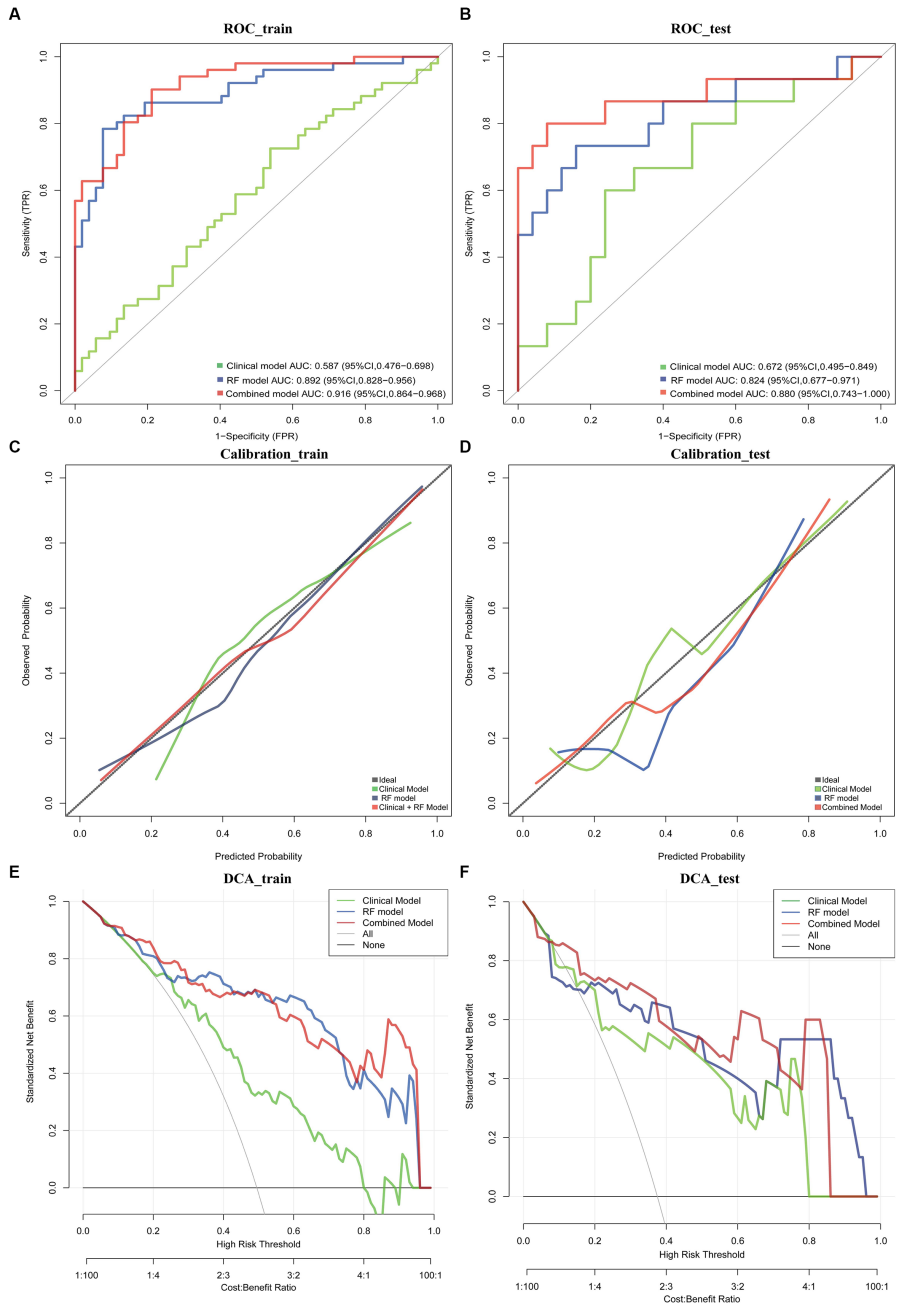
ROC curve in the training set and validation sets for the nomogram **(A,B)**. Calibration curves of this nomogram for distinguishing TERT mutations in training set and validation set **(C,D)**. Clinical decision curve for the clinical model, RF model and combined model **(E,F)**.

The results of the cox proportional hazard regression model are showed in Supplementary material 5. The prognostic factors that influence the survival of patients with GBM, such as age, KPS, and Rad-score, should be considered in the nomogram analysis. Thus, the radiomics nomogram, incorporating the Rad-score along with relevant clinical variables, is presented in Figure 3 to provide a comprehensive prediction of TERT subtypes in patients with GBM. The performance of clinical model, RF model and combined model are showed in Table 3. The results revealed that the combined model yielded a higher diagnostic efficiency than single models in the training and validation sets (Figures 4A,B). The AUC of the nomogram was 0.916 (95% CI, 0.864–0.968) in the training set, 0.880 (95% CI, 0.743–1.000) in the validation set. Delong test revealed that the AUC values in the combined model was significantly higher than that in clinical model (*p* < 0.05). By comparing the predicted outcomes with the actual outcomes, the calibration curve of the nomogram showed good agreement between the TERT subtypes predicted by the radiomics and the actual pathological results (Figures 4C,D). The Hosmer-Lemeshow test further verifies that the goodness-of-fit in the training and validation sets (*p* = 0.369 and 0.284, respectively). On the other hand, DCA of the nomogram is demonstrated by plotting threshold probabilities on the x-axis and net benefit on the y-axis. The blue line represents the decision curve of the RF model. The green line represents the clinical model curve, whereas the red line represents the decision curve of the RF model combined with clinical model of patients with GBM. The combined nomogram model (Figures 4E,F) demonstrated superior performance in predicting TERT mutation status compared to clinical and RF models.

**Table 3:** The AUC, 95% CI, cutoff, accuracy, sensitivity, specificity, PPV, and NPV for the clinical model, RF model and combined model in the training set and validation set.

Models	AUC	95% CI	Cutoff	Acc	Sen	Spe	PPV	NPV	Task
Clinical model	0.587	[0.476–0.698]	0.432	0.709	0.706	0.712	0.706	0.712	Training
	0.672	[0.495–0.849]	0.432	0.775	0.867	0.720	0.650	0.900	Validation
RF model	0.892	[0.828–0.956]	0.560	0.854	0.784	0.923	0.909	0.814	Training
	0.824	[0.677–0.971]	0.560	0.775	0.733	0.800	0.688	0.833	Validation
Combined model	0.916	[0.864–0.968]	0.487	0.845	0.804	0.885	0.872	0.821	Training
	0.880	[0.743–1.000]	0.487	0.930	0.800	0.760	0.684	0.905	Validation

RF, random forest; AUC, the area under the receiver operator characteristics curve, CI, confidence interval, PPV, positive predictive value, NPV, negative predictive value.

### Prognostic performance of the prediction model

**Figure 5:**
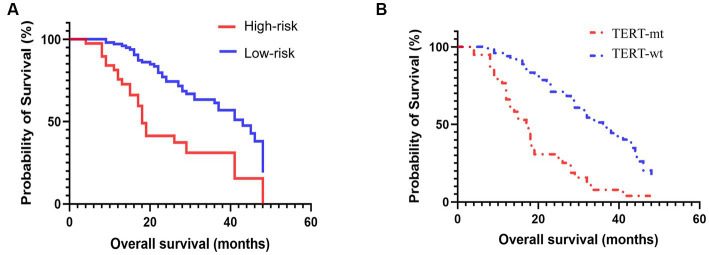
Prognosis based on the risk-stratified based on radiomics signature **(A)** and pathologically confirmed TERT promoter mutation status **(B)**.

Among the 152 patients included in our cohort, The prognostic study comprised 143 patients who were known to have survived until the expiration date or had a specific time of death; the median follow-up duration was 36.8 months. we compared the survival curves of the high-risk and low-risk groups classified by radiomics nomogram with the TERT mutation group and the wild-type group based on pathological diagnosis. The results are shown in Table 4 and Figure 5. According to the radiomics nomogram, patients with a risk score higher than 60.979 were divided into the high-risk subgroup, and patients with a risk score lower than 60.979 were divided into the low-risk subgroup. Subsequently, Kaplan–Meier curves revealed significant differences between the two risk-stratified groups, which was in good agreement with the results of survival analyses in the TERT mutant group (both *p* < 0.001). This finding suggested that patients classified as high-risk exhibited an increased susceptibility to TERT mutation events, whereas patients classified as low-risk demonstrated a lower likelihood of acquiring TERT mutations. However, there was no significant difference between TERT mutant and high-risk subgroups and between TERT wild-type and low-risk subgroups (*p* = 0.322 and *p* = 0.068, respectively). The hazard ratio (HR) for the high-risk group compared to the low-risk group was 2.828 (95% CI, 1.463–5.465), whereas the TERT mutant group exhibited a hazard ratio of 3.267 (95% CI, 1.853–5.762) when compared to the wild-type group. The median survival time of the high-risk group compared to the low-risk group was 18 months versus 43 months, while for the TERT mutation group compared to the wild-type group it was 17 months versus 36 months. The low-risk group and TERT wild type GBM demonstrated a significantly prolonged mean median survival time, indicating a positive impact on overall survival.

**Table 4:** The Kaplan–Meier curve of the pathologically confirmed TERT mutation group and the risk-stratified group.

Groups	Subgroups	Median survival (months)	HR 95% CI	*p*
	TERT-mt	17		
TERT mutation				3.267 [1.853–5.762]
<0.001				
	TERT-wt	36		
	High-risk	18		
Risk stratification				2.828 [1.463–5.465]
<0.001				
	Low-risk	43		

TERT, telomerase reverse transcriptase; HR, hazard ratio; CI, confidence interval.

## Discussion

We herein investigated the relationship between multi-parameter MRI features and the TERT mutation status, established a radiomics nomogram for predicting the TERT promoter mutation status, and verified its efficacy in prognostic assessment of GBM patients. The results revealed that the RF algorithm performed the best, with seven features yielding the highest diagnostic efficiency. Compared with RF models and clinical models, the combined model (clinical features and rad-score based on RF) showed the highest diagnostic efficacy in training and validation sets. The calibration curve and DCA further validate the pivotal clinical role of the nomogram in distinguishing TERT subtypes in GBM patients. Furthermore, we observed consistent alignment between the survival analyses in the risk-stratified group based on nomogram and those observed in the TERT promoter mutant group confirmed through pathological examination. Consequently, our findings suggested that the radiomics nomogram could non-invasively predict TERT subtypes and prognosis in GBM with excellent identification and calibration abilities.

Recently, there has been increasing interest in utilizing radiomics based on multi-parameter MRI for the prediction of TERT mutations in gliomas. One radiomics research used CE-T1WI, FLAIR, and ADC sequences to detect IDH-mutant TERT promoter-mutant gliomas (grades 2–4) and found that the implemented model had an AUC of 0.971 (95% CI: 0.902–1.000), sensitivity of 0.833 (95% CI, 0.333–1.000), and specificity of 0.966 (95% CI, 0.931–1.000) in the test set (23). Park et al. developed a model that incorporated Visually Accessible Rembrandt Images and radiomics characteristics and showed improved performance (AUC = 0.854) in distinguishing TERT mutation of IDH wild-type low-grade gliomas (24). Although multi-parametric radiomics has demonstrated promising results in accurately and sensitively predicting TERT mutations in gliomas, previous studies have primarily focused on grades 2–4 gliomas with limited investigation into GBM. Only two studies (25, 26) highlighted the potential of radiomics to predict TERT promoter mutation status in patients with GBM, providing evidence that radiomic features extracted from routine preoperative MRI images can be used as non-invasive biomarkers for TERT classification. However, it should be noted that these studies do not explicitly address the implementation of the radiomics nomogram. Hence, additional investigation is necessary to examine the prospective utility of incorporating a radiomics nomogram in this particular context.

Radiomics nomogram is a visual representation of the result of logistic regression or Cox regression. It sets a scoring scale based on the size of the regression coefficients for all independent variables, assigning a score to each value level of these variables. By calculating the total score for each patient, the conversion function between the score and the probability of the outcome occurring is used to determine the likelihood of the event time occurring. Studies have shown that the nomogram can offer valuable insights into its diagnostic capabilities and potential applications in clinical practice (17, 27–29). In this study, we systematically selected the most optimal classifier to achieve our objective of providing a more precise and reliable prediction of TERT typing. The ROC curve analysis revealed that the AUC values for RF algorithm in the training set were 0.892, indicating a superior discriminative ability compared to SVM and Lasso, despite all three being widely used machine learning algorithms for classification and regression tasks. Importantly, when we integrated the clinical features and rad-score on the basis of RF, the nomogram for the combined model demonstrated superior performance in predicting TERT mutation status compared to clinical and RF models (the AUC increased from 0.892 to 0.916). Additionally, both the calibration curve and clinical decision curve provide objective measures to evaluate the clinical application value of the nomogram in differentiating TERT subtypes for GBM patients.

In the present study, we also compared the survival curves of the high-risk and low-risk groups as classified by radiomics nomogram with those of the TERT mutation group and the wild-type group based on pathological diagnosis. The findings showed a robust concordance between the predictive efficacy of radiomics and pathological diagnosis for evaluating TERT mutations. Furthermore, the low-risk group and TERT wild-type GBM demonstrated a significantly prolonged mean median survival time, indicating a positive impact on overall survival. The survival analysis further demonstrated a significant association between the presence of TERT promoter mutations and shorter overall survival (OS) when compared to their absence, consistent with the observation of shorter survival in the high-risk group as opposed to the low-risk group. The findings of this study suggested the remarkable predictive capability of radiomics nomogram in identifying TERT mutations and its effectiveness in prognostic assessment, which aligned with previous studies (18, 30, 31). Therefore, we deduce that the incorporation of radiomics signature into clinical decision-making has the potential to be helpful in predicting patient outcomes, particularly during the follow-up in patients who are unable to undergo surgery.

Our current findings also suggest that TERT mutations are related to age, and that the mutation rate of TERT is higher in the elderly population, which was in lined with previous studies (17, 30). Researches have reported varying probabilities of the presence of these mutations, with some studies reporting that these mutations occur in 60–80% of patients with GBM (32–34). In our cohort, TERT mutation was observed in 47.37% patients, which was similar to a study with a mutation rate of 48.27% (35). Theoretically, TERT mutations are hypothesized to augment telomerase activity, thereby facilitating cellular immortalization and potentiation of tumorigenesis. TERT promoter mutations serve as a pivotal oncogenic driver in GBM, facilitating telomerase activation and conferring unimpeded tumor growth and progression (34). The presence of these mutations signifies that the cancer cells have acquired a mechanism to evade the natural limitations set by the cell’s life cycle, enabling them to divide indefinitely and leading to rapid tumor growth (36). Furthermore, the TERT promoter mutations contribute to the aggressive nature of GBM by allowing the cancer cells to develop resistance to treatment, leading to a higher risk of relapse and reduced survival rates (37).

The identification of prognostic biomarkers holds immense significance in the medical field. Despite the generally poor prognosis for GBM patients, individualized treatments based on biomarkers have significantly improved survival rates for certain individuals. By providing a non-invasive and cost-effective method for predicting TERT typing, the radiomics may help to optimize treatment strategies and improve patient outcomes. More importantly, nomogram will help to verify the reliability, accuracy and practicability of the prediction model in clinical settings, and provide a valuable basis for the adoption and implementation of the model. This personalized approach may lead to improved patient outcomes by optimizing the balance between treatment efficacy and potential side effects (38). In future studies, we aim to develop more sophisticated and precise machine learning models to enhance the accuracy and robustness of TERT classification. Furthermore, integration of image data from different modalities such as structural MRI, functional MRI, and PET will be employed to improve the precision and reliability of TERT typing.

Our study has some limitations. First, although all patients included were from two institutions (the sample size of the center II was too small to be used as an external validation cohort), further validation and optimization of these models on larger, more diverse patient cohorts are needed to uphold our results. Second, we used common MRI sequences to construct the radiomics signature. Although our results showed good diagnostic performance, incorporating more advanced MRI techniques is needed to improve discriminative abilities. Furthermore, TERT gene expression may vary between different regions of the same tumor, which may lead to unreliable diagnostic results. Hence, Advanced imaging techniques could be used to identify the most representative areas of the tumor for biopsy.

## Conclusion

The implementation of the RF algorithm can improve the diagnostic performance for detecting TERT mutations in patients with GBM. Moreover, the radiomics nomogram constructed by integrating RF algorithm and clinical features exhibits superior performance for determining TERT mutation status, thereby serving as an optimal decision-making tool to maximize net benefit in prognosis prediction. This approach holds great promise for optimizing treatment strategies and improving patient outcomes in future endeavors.

## Data availability statement

The original contributions presented in the study are included in the article/Supplementary material, further inquiries can be directed to the corresponding authors.

## Ethics statement

The studies involving humans were approved by the Ethics Committee of Guangxi Medical University. The studies were conducted in accordance with the local legislation and institutional requirements. The ethics committee/institutional review board waived the requirement of written informed consent for participation from the participants or the participants’ legal guardians/next of kin because this is a retrospective study, so no informed consent is required.

## Author contributions

LC: Funding acquisition, Writing – original draft, Writing – review & editing. RC: Project administration, Writing – review & editing. TL: Formal analysis, Funding acquisition, Resources, Investigation, Writing – review & editing. CT: Data curation, Software, Validation, Visualization, Formal analysis, Writing – review & editing. YL: Conceptualization, Methodology, Project administration, Supervision, Writing – review & editing. ZZ: Conceptualization, Project administration, Supervision, Methodology, Validation, Writing – review & editing.

## Funding

The author(s) declare financial support was received for the research, authorship, and/or publication of this article. This study was partially supported by grants from Guangxi medical and health appropriate technology development and application project (S201670), Guangxi Liuzhou Science and Technology Planning Project (2021CBC0128), Guangxi Zhuang Autonomous Region self-funded project (Z20210919).

## Conflict of interest

The authors declare that the research was conducted in the absence of any commercial or financial relationships that could be construed as a potential conflict of interest.

## Publisher’s note

All claims expressed in this article are solely those of the authors and do not necessarily represent those of their affiliated organizations, or those of the publisher, the editors and the reviewers. Any product that may be evaluated in this article, or claim that may be made by its manufacturer, is not guaranteed or endorsed by the publisher.

## Footnotes

^1^https://www.slicer.org/

^2^https://github.com/salan668/FAE

## References

[1] Gimple RC Bhargava S Dixit D Rich JN. Glioblastoma stem cells: lessons from the tumor hierarchy in a lethal cancer. Genes Dev. (2019) 33:591–609. doi: 10.1101/gad.324301.119, PMID: 31160393 PMC6546059

[2] Omuro A DeAngelis LM. Glioblastoma and other malignant gliomas: a clinical review. JAMA. (2013) 310:1842–50. doi: 10.1001/jama.2013.280319, PMID: 24193082

[3] Louis DN Perry A Wesseling P Brat DJ Cree IA Figarella-Branger D. The 2021 WHO classification of Tumors of the central nervous system: a summary. Neuro Oncol. (2021) 23:1231–51. doi: 10.1093/neuonc/noab106, PMID: 34185076 PMC8328013

[4] Smith HL Wadhwani N Horbinski C. Major features of the 2021 WHO classification of CNS Tumors. Neurotherapeutics. (2022) 19:1691–704. doi: 10.1007/s13311-022-01249-0, PMID: 35578106 PMC9723092

[5] Gritsch S Batchelor TT Gonzalez Castro LN. Diagnostic, therapeutic, and prognostic implications of the 2021 World Health Organization classification of tumors of the central nervous system. Cancer. (2022) 128:47–58. doi: 10.1002/cncr.33918, PMID: 34633681

[6] Olympios N Gilard V Marguet F Clatot F Di Fiore F Fontanilles M. TERT promoter alterations in glioblastoma: a systematic review. Cancers. (2021) 13:1147. doi: 10.3390/cancers13051147, PMID: 33800183 PMC7962450

[7] Śledzińska P Bebyn MG Furtak J Kowalewski J Lewandowska MA. Prognostic and predictive biomarkers in gliomas. Int J Mol Sci. (2021) 22:10373. doi: 10.3390/ijms221910373, PMID: 34638714 PMC8508830

[8] Le Rhun E Preusser M Roth P Reardon DA van den Bent M Wen P. Molecular targeted therapy of glioblastoma. Cancer Treat Rev. (2019) 80:101896. doi: 10.1016/j.ctrv.2019.101896, PMID: 31541850

[9] Smith EM Pendlebury DF Nandakumar J. Structural biology of telomeres and telomerase. Cell Mol Life Sci. (2020) 77:61–79. doi: 10.1007/s00018-019-03369-x, PMID: 31728577 PMC6986361

[10] Miki S Koga T McKinney AM Parisian AD Tadokoro T Vadla R. TERT promoter C228T mutation in neural progenitors confers growth advantage following telomere shortening *in vivo*. Neuro Oncol. (2022) 24:2063–75. doi: 10.1093/neuonc/noac080, PMID: 35325218 PMC9713509

[11] Tahara H Yasui W Tahara E Fujimoto J Ito K Tamai K. Immuno-histochemical detection of human telomerase catalytic component, hTERT, in human colorectal tumor and non-tumor tissue sections. Oncogene. (1999) 18:1561–7. doi: 10.1038/sj.onc.1202458, PMID: 10102626

[12] Matsumura N Nakajima N Yamazaki T Nagano T Kagoshima K Nobusawa S. Concurrent TERT promoter and BRAF V600E mutation in epithelioid glioblastoma and concomitant low-grade astrocytoma. Neuropathology. (2017) 37:58–63. doi: 10.1111/neup.12318, PMID: 27302309

[13] Horn S Figl A Rachakonda PS Fischer C Sucker A Gast A. TERT promoter mutations in familial and sporadic melanoma. Science. (2013) 339:959–61. doi: 10.1126/science.1230062, PMID: 23348503

[14] Hooper GW Ginat DT. MRI radiomics and potential applications to glioblastoma. Front Oncol. (2023) 13:1134109. doi: 10.3389/fonc.2023.1134109 36874083 PMC9982088

[15] Wang J Yi X Fu Y Pang P Deng H Tang H. Preoperative magnetic resonance imaging radiomics for predicting early recurrence of glioblastoma. Front Oncol. (2021) 11:769188. doi: 10.3389/fonc.2021.769188, PMID: 34778086 PMC8579096

[16] Jia X Zhai Y Song D Wang Y Wei S Yang F. A multiparametric MRI-based radiomics nomogram for preoperative prediction of survival stratification in glioblastoma patients with standard treatment. Front Oncol. (2022) 12:758622. doi: 10.3389/fonc.2022.758622, PMID: 35251957 PMC8888684

[17] Tian H Wu H Wu G Xu G. Noninvasive prediction of TERT promoter mutations in high-grade glioma by radiomics analysis based on multiparameter MRI. Biomed Res Int. (2020) 2020:3872314. doi: 10.1155/2020/3872314, PMID: 32509858 PMC7245686

[18] Yan J Zhang B Zhang S Cheng J Liu X Wang W. Quantitative MRI-based radiomics for noninvasively predicting molecular subtypes and survival in glioma patients. NPJ Precis Oncol. (2021) 5:72. doi: 10.1038/s41698-021-00205-z 34312469 PMC8313682

[19] Fukuma R Yanagisawa T Kinoshita M Shinozaki T Arita H Kawaguchi A. Prediction of IDH and TERT promoter mutations in low-grade glioma from magnetic resonance images using a convolutional neural network. Sci Rep. (2019) 9:20311. doi: 10.1038/s41598-019-56767-3 31889117 PMC6937237

[20] Arita H Narita Y Fukushima S Tateishi K Matsushita Y Yoshida A. Upregulating mutations in the TERT promoter commonly occur in adult malignant gliomas and are strongly associated with total 1p19q loss. Acta Neuropathol. (2013) 126:267–76. doi: 10.1007/s00401-013-1141-6, PMID: 23764841

[21] Fang S Fan Z Sun Z Li Y Liu X Liang Y. Radiomics features predict telomerase reverse transcriptase promoter mutations in World Health Organization grade II gliomas via a machine-learning approach. Front Oncol. (2020) 10:606741. doi: 10.3389/fonc.2020.606741, PMID: 33643908 PMC7905226

[22] Wang P Tang Z Xiao Z Hong R Wang R Wang Y. Dual-energy CT in differentiating benign sinonasal lesions from malignant ones: comparison with simulated single-energy CT, conventional MRI, and DWI. Eur Radiol. (2022) 32:1095–105. doi: 10.1007/s00330-021-08159-3, PMID: 34427744

[23] Wang H Zhang S Xing X Yue Q Feng W Chen S. Radiomic study on preoperative multi-modal magnetic resonance images identifies IDH-mutant TERT promoter-mutant gliomas. Cancer Med. (2023) 12:2524–37. doi: 10.1002/cam4.5097, PMID: 36176070 PMC9939206

[24] Park CJ Han K Kim H Ahn SS Choi D Park YW. MRI features may predict molecular features of glioblastoma in isocitrate dehydrogenase wild-type lower-grade gliomas. AJNR Am J Neuroradiol. (2021) 42:448–56. doi: 10.3174/ajnr.A6983, PMID: 33509914 PMC7959428

[25] Zhang H Zhang H Zhang Y Zhou B Wu L Lei Y. Deep learning radiomics for the assessment of telomerase reverse transcriptase promoter mutation status in patients with glioblastoma using multiparametric MRI. J Magn Reson Imaging. (2023). doi: 10.1002/jmri.28671 [Epub ahead of print]., PMID: 36896953

[26] Calabrese E Rudie JD Rauschecker AM Villanueva-Meyer JE Clarke JL Solomon DA. Combining radiomics and deep convolutional neural network features from preoperative MRI for predicting clinically relevant genetic biomarkers in glioblastoma. Neurooncol Adv. (2022) 4:vdac060. doi: 10.1093/noajnl/vdac060, PMID: 35611269 PMC9122791

[27] Hamada T Nakai Y Yasunaga H Isayama H Matsui H Takahara N. Prognostic nomogram for nonresectable pancreatic cancer treated with gemcitabine-based chemotherapy. Br J Cancer. (2014) 110:1943–9. doi: 10.1038/bjc.2014.131, PMID: 24642625 PMC3992497

[28] Etcheverry A Aubry M Idbaih A Vauleon E Marie Y Menei P. DGKI methylation status modulates the prognostic value of MGMT in glioblastoma patients treated with combined radio-chemotherapy with temozolomide. PLoS One. (2014) 9:e104455. doi: 10.1371/journal.pone.0104455, PMID: 25233099 PMC4169423

[29] Kim DY Shim SH Kim SO Lee SW Park JY Suh DS. Preoperative nomogram for the identification of lymph node metastasis in early cervical cancer. Br J Cancer. (2014) 110:34–41. doi: 10.1038/bjc.2013.718, PMID: 24231954 PMC3887306

[30] Lu J Li X Li H. A radiomics feature-based nomogram to predict telomerase reverse transcriptase promoter mutation status and the prognosis of lower-grade gliomas. Clin Radiol. (2022) 77:e560–7. doi: 10.1016/j.crad.2022.04.005, PMID: 35595562

[31] Park YW Kim S Park CJ Ahn SS Han K Kang SG. Adding radiomics to the 2021 WHO updates may improve prognostic prediction for current IDH-wildtype histological lower-grade gliomas with known EGFR amplification and TERT promoter mutation status. Eur Radiol. (2022) 32:8089–98. doi: 10.1007/s00330-022-08941-x, PMID: 35763095

[32] Lötsch D Ghanim B Laaber M Wurm G Weis S Lenz S. Prognostic significance of telomerase-associated parameters in glioblastoma: effect of patient age. Neuro Oncol. (2013) 15:423–32. doi: 10.1093/neuonc/nos329, PMID: 23393205 PMC3607268

[33] Mosrati MA Malmström A Lysiak M Krysztofiak A Hallbeck M Milos P. TERT promoter mutations and polymorphisms as prognostic factors in primary glioblastoma. Oncotarget. (2015) 6:16663–73. doi: 10.18632/oncotarget.4389, PMID: 26143636 PMC4599297

[34] Giunco S Padovan M Angelini C Cavallin F Cerretti G Morello M. Prognostic role and interaction of TERT promoter status, telomere length and MGMT promoter methylation in newly diagnosed IDH wild-type glioblastoma patients. ESMO Open. (2023) 8:101570. doi: 10.1016/j.esmoop.2023.101570, PMID: 37230028 PMC10265608

[35] Jiang C Kong Z Zhang Y Liu S Liu Z Chen W. Conventional magnetic resonance imaging-based radiomic signature predicts telomerase reverse transcriptase promoter mutation status in grade II and III gliomas. Neuroradiology. (2020) 62:803–13. doi: 10.1007/s00234-020-02392-1, PMID: 32239241

[36] Li X Qian X Wang B Xia Y Zheng Y Du L. Programmable base editing of mutated TERT promoter inhibits brain tumour growth. Nat Cell Biol. (2020) 22:282–8. doi: 10.1038/s41556-020-0471-6, PMID: 32066906

[37] Heidenreich B Rachakonda PS Hosen I Volz F Hemminki K Weyerbrock A. TERT promoter mutations and telomere length in adult malignant gliomas and recurrences. Oncotarget. (2015) 6:10617–33. doi: 10.18632/oncotarget.3329, PMID: 25797251 PMC4496380

[38] Simon M Hosen I Gousias K Rachakonda S Heidenreich B Gessi M. TERT promoter mutations: a novel independent prognostic factor in primary glioblastomas. Neuro Oncol. (2015) 17:45–52. doi: 10.1093/neuonc/nou158, PMID: 25140036 PMC4483052

