# Supplementary material for: Multi-parameter MRI based radiomics nomogram for predicting telomerase reverse transcriptase promoter mutation and prognosis in glioblastoma
Source: Front Neurol. 2023 Sep 26;14:1266658. doi: 10.3389/fneur.2023.1266658 (PMC10565857; doi:10.3389/fneur.2023.1266658)
Supplement: Supplementary file 1 [file Data_Sheet_1.docx]

**Supplementary 1**: Magnetic Resonance Imaging Protocol.

| **parameter** | **3.0 T Philips**  **(n=64)** | **1.5 T Siemens**  **(13)** | **1.5 T GE**  **(19)** | **3.0 T GE**  **(89)** |
| --- | --- | --- | --- | --- |
| **T2WI** | | | | |
| TR/TE (ms) | 2600/80 | 4950/104 | 4460/126.2 | 5844/129 |
| FOV | 512*512 | 378*448 | 512*512 | 512*512 |
| FA (^。^) | 90 | 90 | 90 | 90 |
| Matrix | 256*217 | 256*203 | 512*192 | 256*217 |
| Slice thickness/gap (mm) | 5/3 | 5/2.5 | 5/2.5 | 5/2.5 |
| **DWI** | | | | |
| TR/TE (ms) | 2429/81 | 4850/81 | 5500/99.4 | 2155/61.2 |
| FOV | 192*192 | 196*196 | 256*256 | 256*256 |
| FA (^。^) | 90 | 90 | 90 | 90 |
| Matrix | 120*127 | 256*203 | 128*128 | 256*217 |
| Slice thickness/gap (mm) | 5/1 | 5/1 | 5/2.5 | 5/1 |

**
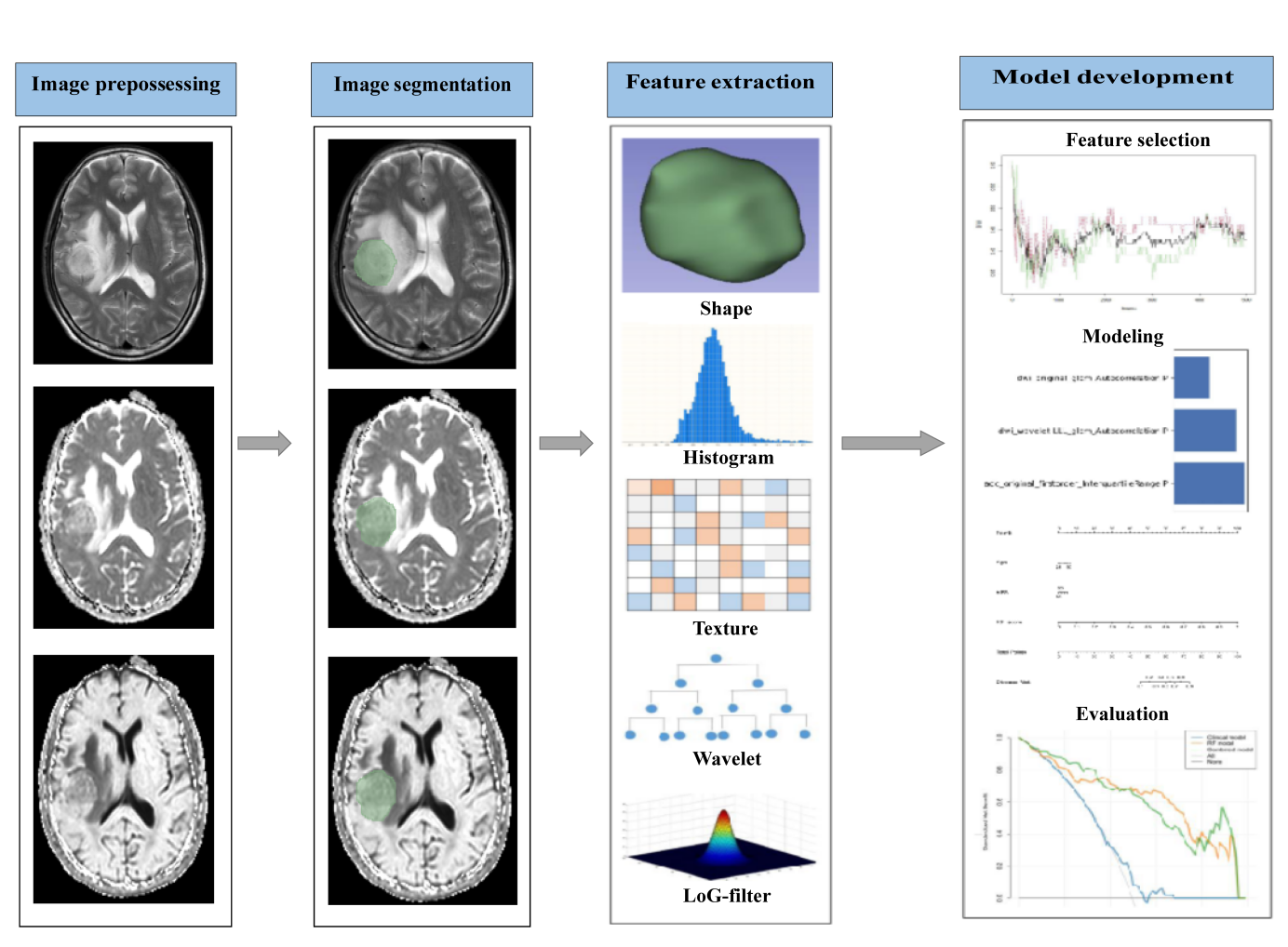
**

**Supplementary 2**: Radiomics processing flow.

**Supplementary 3**: The classification and parameter composition of radiomics features.

| **Feature classifier Feature parameters (n=944)** |
| --- |
| Shape feature (n=14)  Elongation, Flatness, Least axis length, Major axis length, Maximum 2D diameter column, Maximum 2D diameter row, Maximum 2D diameter Slice, Maximum 3D diameter, Mesh volume, Minor axis length, Sphericity, Surface area, Surface volume ratio, Voxel volume  Histogram feature (n=18)  P10, P90, Interquartile range, Energy, Entropy, Skewness, Kurtosis, Maximum, Minimum, Mean, Mean absolute, Deviation, Median, Total energy, Uniformity, Variance, Range, Robust mean absolute deviation, Root mean squared  Texture feature (n=75)  Grey-level co-occurrence matrix, GLCM(n=24); grey-level dependence matrix, GLDM(n=14); grey-level run length matrix, GLRLM(n=16); grey-level size zone matrix, GLSZM(n=16); neighborhood gray-tone difference matrix, NGTDM (n=5)  Wavelet transform (n=744)  Wavelet filtering produces 8 decomposition per stage. In the three dimensions, all feasible combinations of high-pass or low-pass filters (LLH, LHL, LHH, HLL, HLH, HHL, HHH, LLL)  LoG filter (n=93)  Sigma-1-mm-3D-firstorder(n=18), Sigma-1-mm-3D-glcm(n=24), Sigma-1-mm-3D-gldm(n=14), Sigma-1-mm-3D-glrlm(n=16), Sigma-1-mm-3D-glszm(n=16), Sigma-1-mm-3D-ngtdm(n=5) |

**
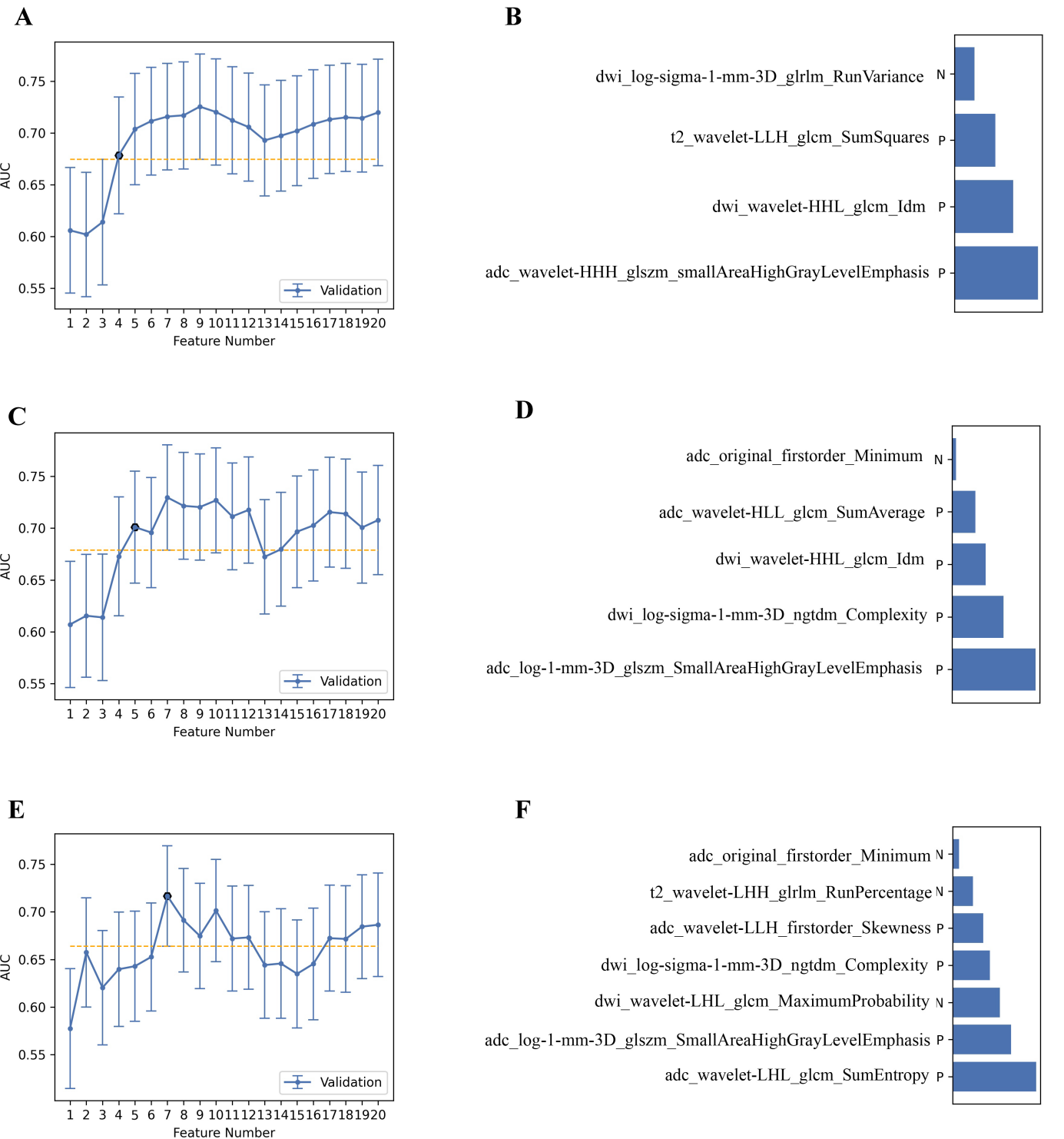
**

**Supplementary 4:** SVM, LASSO and RF were used to screen the radiomics features, and the10-fold cross validation method was used to screen the feature sets with the best performance. A: SVM model with 13 features yielded the highest diagnostic efficiency, and the 4 most important features were chosen by the “1-SE” principle; B: The parameters that contributed the most to TERT mutation discrimination. C: LASSO model with 15 features yielded the highest diagnostic efficiency, and the 5 most important features were chosen by the “1-SE” principle; D: The parameters that contributed the most to TERT mutation discrimination. E: RF model with 20 features yielded the highest diagnostic efficiency, and the 7 most important features were chosen by the “1-SE” principle; F: The parameters that contributed the most to TERT mutation discrimination.

**Supplementary 5:** The result for age, gender, KPS score and RF rad-score by cox proportional hazard regression model.

| **Variables B HR 95%CI P** |
| --- |
| Age 0.024 1.032 0.62-1.74 0.042  Gender -2.223 0.605 0.29-1.73 0.305  KPS -0.015 1.374 0.62-3.98 0.029  Rad-score 1.114 2.131 1.45-6.37 0.003 |

RF: Random Forest; KPS: Karnofsky performance status. HR: hazard ratio; CI: confidence interval.

Multivariate COX regression analysis showed that age, KPS score and rad-score were independent prognostic factors to distinguish TERT promoters in GBM patients. Therefore, we used these factors to construct a nomogram to predict TERT mutation and verify its efficacy in prognosis in GBM patients.

**The formula for calculating the Cox regression equation :**

Equation=0.024*Age+-0.015*KPS+1.114*Rad-score
